# The Effect of Early Neurological Stimulation on Puppy Welfare in Commercial Breeding Kennels

**DOI:** 10.3390/ani13010071

**Published:** 2022-12-24

**Authors:** Grace Boone, Aynsley C. Romaniuk, Shanis Barnard, Traci Shreyer, Candace Croney

**Affiliations:** Department of Comparative Pathobiology, Purdue University, West Lafayette, IN 47907, USA

**Keywords:** breeding dogs, ENS, ground transportation, canine welfare, early gentling, puppy development

## Abstract

**Simple Summary:**

Dogs may experience many stressful situations throughout their lives. Studies suggest that gentle handling in early life may help animals better cope with stressors as adults, but studies in dogs are inconclusive. This study applied gentle early handling (i.e., early neurological stimulation or ENS) to puppies during the first four weeks of life and compared welfare and developmental metrics with two control groups (non-handled and hold-only). A total of 76 puppies from one commercial breeding kennel were assessed. Puppies’ physical health was measured for the first eight weeks of their lives. Their behavioral responses to mild stressors (i.e., isolation and stranger approach tests) were measured at approximately 8 weeks old, before and after ground transportation to a distributor. Puppies were generally physically healthy and clean. Puppies showed more affiliative responses to the stranger-approach test after the 3-minute isolation test than before, independent of the treatment group. Overall, findings suggest that for puppies in commercial breeding kennels, ENS may not be more beneficial in modifying their stress responses than consistent, careful, general handling.

**Abstract:**

Throughout their lives, dogs may experience various stressful events. Early neurological stimulation (ENS), which was shown to alter stress responses beneficially in some animals, has not been fully explored in dogs. Seventy-six small-breed puppies from one commercial breeding kennel were divided into three treatment groups: ENS, held, and control. Puppies in the ENS group received 30 s of handling exercises for 21 days after birth; puppies in the held group were simply held for the same amount of time. Puppies in the control group were managed as per normal breeder practices (i.e., routine husbandry and physical health checks). Physical health was assessed weekly, and puppies were generally healthy and clean. Behavioral responses to stranger approach and isolation tests were evaluated pre- and post-ground-transportation to a distributor. Puppies were more affiliative toward a stranger post-isolation than pre-isolation (*p* < 0.001), and post-transport than pre-transport (*p* < 0.001). At the distributor, puppies in the isolation test spent less time in exploratory locomotion (*p* < 0.001) and vocalized more than at the breeder’s kennel (*p* = 0.011). Treatment did not affect these results. Overall, the results suggest that the type of ENS used in this study may not provide the purported benefits to puppies’ stress responses in commercial breeding populations.

## 1. Introduction

Dogs and puppies in commercial breeding (CB) kennels may be exposed to many potentially stressful situations common to kennel environments, including high noise levels, limited human interaction, lack of environmental enrichment, and social isolation [1]. Puppies from CB kennels are likely to undergo additional stressors, including weaning and ground transportation to a distributor or pet store [2]. An animal’s response to stress can vary in type and intensity according to their perception of the stressor, and on factors such as genetics, temperament, and previous experience [3]. A sustained stress response may have widespread physical and behavioral effects on the individual, such as immunosuppression [4,5] and cognitive impairments [6,7,8]. Adverse experiences can influence an organism at any age or developmental stage but may have an especially profound effect during early life. Not only is early life a period of heightened development, but it is also suggested that early life experiences inform the programming of certain functions, such as the stress response, as the organism uses cues present in the early environment to predict what the future environment will be and adapt accordingly [9,10,11]. 

Applying gentle stressors to an animal during its critical early developmental period may result in an “inoculation effect” via changes to the animal’s behavioral and neurological development that enable better coping with stressors experienced later in life. Handling interventions and brief maternal separation protocols (commonly referred to as early neurological stimulation, ENS) have been employed as mild stressors in early life in several species (rats [12,13,14], mice [15], non-human primates [16,17,18], cats [19,20], and pigs [21]). Many of these studies have demonstrated changes which suggest improved modulation of the stress response in handled animals as compared to unhandled controls (for a review, see [22]). For example, studies in rats identified benefits of ENS such as lowered behavioral and physiological reactivity in an open-field test [13]; lower concentrations of the stress hormone corticosterone in response to stress and a quicker return to baseline [14]; and less resistance to capture and/or handling [23]. Furthermore, a recent study of rats that was conducted by Castelli and colleagues [12] found that early handling and a 15-minute separation from days 2 to 21 of life prompted increases in the number of maternal care behaviors measured (pup retrieval, nursing, and licking, among others) and offset the detrimental effects on pups of administering prenatal glucocorticoid to dams. Rat pups who were handled showed increased locomotor activity, more swimming in a forced swim test, and lowered concentrations of plasma corticosterone as compared to unhandled pups [12]. Mechanisms behind these effects may include brief activations of the offspring’s hypothalamic–pituitary–adrenal axis; changes in maternal behavior (i.e., increased licking and arched-back nursing postures) after pups’ return from separation [24,25]; or other factors, such as environmental stress or a drop in body temperature [26,27,28]. Studies of ENS in squirrel monkeys have shown that the effects can potentially last for years [18], while studies in cats have highlighted the interplay between genetics and experience (i.e., ENS) in shaping their responses to stressors, such as willingness to approach and interact with novel humans and objects [19].

While positive effects of neonatal handling have been observed in some studies, effects in others have not always been straightforward. For example, some studies have shown few biological and no behavioral differences between handled and unhandled animals [29]. Others have identified potentially detrimental outcomes of ENS treatment, such as decreased social play behavior [30] and altered reproductive and feeding habits [31,32]. This is especially highlighted in the related literature pertaining to dogs, as relatively little has been published on this type of early handling, and results are inconclusive. To illustrate, an early study on the effects of ENS in dogs employed an hour of various types of stimulation applied daily for the first five weeks of life to three treatment groups: handled puppies, controls, and partially socially isolated puppies [33]. Puppies were then tested at five weeks of age, using a 15-minute arena test with objects. Handled puppies were found to be generally highly exploratory, social toward humans, and better at problem-solving in barrier tasks than unhandled or partially socially isolated puppies. However, they produced the most vocalizations after the handler removed objects from the arena [33]. There were no significant differences between treatment groups in body weight, brain weight, or reflexes [33].

After this early study was conducted, further interest in puppy ENS was sparked by a review of a protocol developed for military working dogs (MWDs) called “Bio Sensor” [34]. The Bio Sensor program was intended to enhance the ability of puppies to perform as MWDs and included six stimulation exercises (five described in review) applied for 3–5 s each to puppies from days 3 to 16 of life. In this review, Battaglia stated that the military claimed Bio Sensor exercises enhanced dogs’ cardiovascular and adrenal performance and increased their stress tolerance and disease resistance [34]. However, it is not clear from the report what specific metrics were used to make these determinations. The Bio Sensor protocol was further explored by researchers Schoon and Bernsten, who applied it to puppies who were being raised to become mine-detection dogs [35]. A handling control group, in which puppies were simply held for the duration of the ENS exercises to control for the effect of human contact, was added to the ENS and control groups [35]. This study found no significant differences between puppies who received ENS exercises and handled controls on any of the developmental parameters measured. The authors attributed the lack of effects of ENS on puppies in this study in part to the rich socialization program already in place, which spanned the first 10 weeks of life [35]. The interaction effect between environment and ENS was also advanced by Gazzano and colleagues, who hypothesized, based on their results, that the effects of ENS may be more apparent when puppies are raised in a relatively barren environment, as ENS likely effects greater change in these puppies’ daily lives than it does for puppies raised in an enriched environment [36].

More recently, Gazit, Terkel, and Goldblatt studied the Bio Sensor ENS protocol on Malinois puppies being raised to be MWDs [37]. They found differences between ENS and control-group puppies in odor search motivation and aggression (biting and attack drive) but noted that significant differences between groups were not apparent until 10 to 12 months of age [37]. They suggested that part of the reason for the differences between groups could have been because caretakers were not blind to the treatments, potentially leading to unconscious bias during their training that resulted in puppies in the ENS group being provided with more attention [37].

Given the inconsistencies in findings to date and the small number of published studies, the efficacy of ENS as a potential intervention to improve welfare and stress resilience of puppies raised in CB kennels warrants further investigation. The aim of the current study was to determine if providing ENS to puppies in CB kennels changed their physical health and/or behavioral responses to stressors (e.g., social isolation and transportation) in ways that could ultimately improve their welfare. The hypothesis was that puppies who received ENS would show differences in their physical health metrics (e.g., less illness and increased weight) and their fear and exploratory behaviors (e.g., more affiliative toward strangers and more exploration of a novel area when alone) as compared to the matched controls.

## 2. Materials and Methods

### 2.1. Ethical Statement

The procedures outlined in this study were reviewed and approved by the Purdue University Institutional Animal Care and Use Committee (protocol #1809001797). A consent form outlining what participation in this study would entail was reviewed and signed by the breeder before the study began. This breeder did not have any early puppy handling protocols in place before his enrollment in this study. Routine management and husbandry were unaltered during the study period. Therefore, all puppies, including those from our control group, were not deprived of any handling/interaction that they would have otherwise received if they were not included in the study.

### 2.2. Subjects and Facilities

One USDA (United States Department of Agriculture) licensed CB kennel, located in Ohio, USA, was utilized for this study. Puppies born between 29 July and 9 September 2019, representing 16 litters and 6 different breeds, were included in the study (Table 1). Only 1 male puppy in the ENS group was scored underweight in his week-one health assessment and died in his second week of life, leaving 76 puppies in the study (36 females and 40 males). These puppies comprised two cohorts, consisting of litters born within about one week of each other (Cohort 1, 5 litters; Cohort 2, 11 litters, average litter size = 4.7, and median = 4). At approximately 8 weeks old, puppies within each cohort were ground transported to a distributor at the same time to allow evaluation of their responses to this common stressor [2]. Puppies born by Cesarean section, or those who were cross-fostered, were excluded from the study population.

Roughly one-third (38.2%) of the puppies in the study population belonged to breeds which traditionally have their ears cropped, which is a legal practice in the USA. As a result, these litters (noted with an asterisk in Table 1) had their ears cropped between two and six weeks of age.

### 2.3. Handling Procedure

Puppies within each litter were assigned to one of three treatment groups (ENS, held, and control, described below), using a balanced random assignment (random UX app for Android). Microchips were not implanted in puppies before 6–7 weeks of age, consistent with normal practices in this kennel. Therefore, all puppies were marked for identification by Researcher 1 (GB), using non-toxic marking pens (Mosaiz, China). To minimize additional handling outside of treatment, the breeder refreshed markings daily during routine husbandry chores, i.e., when puppies were already being handled for health checks or cleaning.

Treatment administration began on day three postpartum and was carried out daily, Monday through Saturday, for 21 days. Testing was not carried out on Sundays due to religious proscriptions at the facility. To minimize stress on dams, all puppies were retrieved from the litter by a familiar handler and then handed to Researcher 1, who applied the treatments described below. A smartphone timer (Google clock app, Google, Mountain View, CA, USA) was used to time the handling treatments. Puppy order for handling within each litter was randomized (Google random-number generator, Google, Mountain View, CA, USA). To protect the puppies and other dogs on-site from disease, strict biosecurity procedures were implemented, such as washing hands before and after puppy handling, sanitizing hands, and wearing new gloves and disposable gowns over clothing between handling of different litters.

#### 2.3.1. ENS Treatment Group

**Figure 1:**
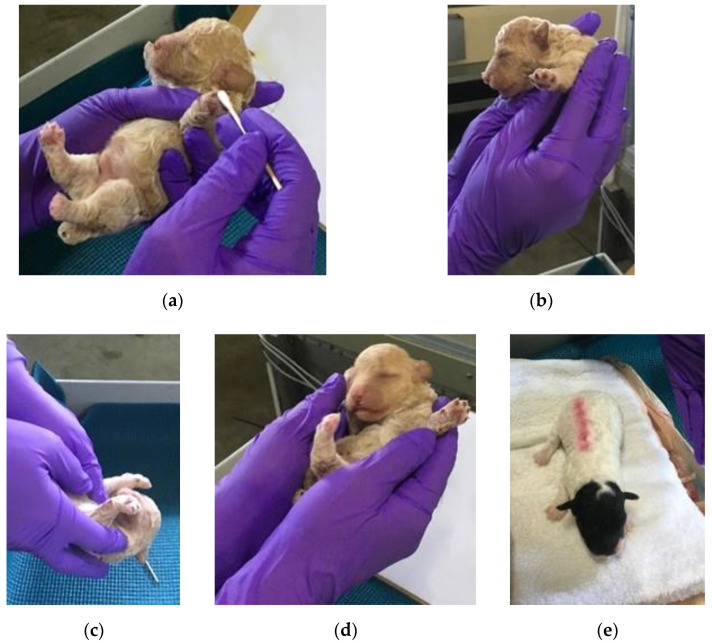
Bio Sensor ENS exercises: (**a**) tactile stimulation—the puppy’s toes were tickled gently with a Q-tip; (**b**) head held erect—the puppy was held so the head is directly above the tail; (**c**) head pointed down—the puppy was held so the tail is above the head; (**d**) supine position—the puppy was held on its back; and (**e**) thermal stimulation—the puppy was placed on a cool, damp washcloth.

The ENS group received five exercises based on the Bio Sensor protocol, as described in Battaglia’s 2009 report [34]. Each exercise was applied for five seconds in the following order: tactile stimulation, head erect, head down, supine, and thermal stimulation (Figure 1).

#### 2.3.2. Held Treatment Group

**Figure 2:**
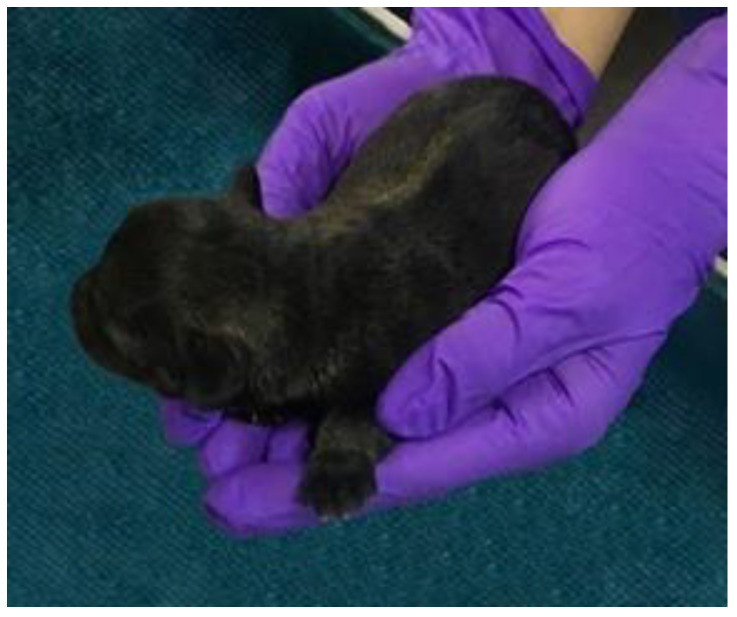
Held treatment—the puppy was held in a sternal position.

Puppies in the held treatment group were held in a sternal position (Figure 2) for 30 s to approximate the amount of handling time received by those in the ENS group. This allowed for evaluation of the effects of ENS exercises beyond those associated with removal from the nest and human handling.

#### 2.3.3. Control Group

Control-group puppies were marked for identification daily, were weighed weekly, and assessed for physical health (detailed below). Otherwise, they received no additional handling besides that in place for regular care and cleaning by the breeder.

### 2.4. Physical Health Assessments

All puppies were weighed within a day of birth by the breeder. Subsequently, researchers weighed puppies and assessed their physical health weekly, from day seven postpartum to eight weeks of age. As part of the PuppyFIDO tool (adapted from the validated FIDO tool for adult dogs [38,39]), puppies’ behavioral responses to stranger approach and physical health were assessed. During the latter, a visual assessment consistent with that described by Romaniuk et al. [2] was performed where puppies were given a body-condition score (BCS), using a 1–3 scale (1 = thin, 2 = normal, and 3 = obese). Furthermore, puppies’ cleanliness was scored by using values denoting the percentage of the body covered in debris (0%, <25%, 26–50%, 51–75%, and >76%) [39]. Nasal and ocular discharge, sneezing, coughing, symptoms of upper respiratory infection, missing fur or poor coat, wounds, sores, lesions, diarrhea, and pyoderma (skin infection) were all recorded as present or absent (Y/N).

Researchers conducted all puppy physical health assessments for cohort one, and through weeks 3–5 for cohort two, after which they were performed by the breeder and known handler, using scoring sheets provided by the researchers. Puppy physical health assessments were also performed on all puppies at eight weeks of age by Researcher 2 (AR), following behavioral testing pre- and post-transport. Inter-rater reliability training was conducted with the breeder, known handler, and the researchers until there was at least 90% agreement on all physical health scores for 10 puppies. All raters were blind to the treatment groups to which puppies were assigned.

### 2.5. Testing Procedures

Puppies underwent ground transportation to a distributor at approximately eight weeks of age. They were assessed in their home kennels 2–3 days pre-transport and again at the distributor approximately 48 h post-transport. For identification purposes, all puppies were collared before testing by the breeder (male) at the kennel or a male researcher at the distributor. Marking was not possible, as puppies needed to be clean for sale. Puppies were given a 30 min acclimation period between collaring and testing. Each puppy was retrieved from the litter by a known caretaker at the kennels and a research assistant at the distributor and individually placed into an isolation pen set up in a quiet area of the facility. To create the isolation pen, a 1 m × 1.5 m surface was covered in black interlocking rubber tiles (Rubber-Cal Armor-Lock), and a metal exercise pen with a locking door (six-61 cm panels, Precision Pet Products) was placed on top. A digital video camera on a tripod was placed near the pen to record the tests for later scoring. Immediately before and after the isolation test, each puppy was scored by Researcher 2, using a brief three-step stranger-approach test (PuppyFIDO test) described below. At the end of the test, physical health was assessed as described previously.

For biosecurity, the isolation pen was spot cleaned in between puppies if soiled and fully disinfected between litters, using Rescue^®^ cleaner (Virox Technologies Inc., Oakville, ON, Canada).

#### 2.5.1. Stranger-Approach Tests

All stranger-approach tests were conducted by an unfamiliar person (Researcher 2) who was blind to the treatment group assignment. The stranger-approach test, i.e., the behavioral portion of the PuppyFIDO (Field Instantaneous Dog Observation) test, was originally outlined by Romaniuk and colleagues [2]. The PuppyFIDO was performed immediately before the isolation test (preISO) and immediately after the isolation time ended (postISO). In brief, the stranger-approach test was conducted as follows:

Step 1: Approach—The unfamiliar person crouched down in front of the pen door, while turned to the side with averted gaze, and scored the puppy’s behavioral response. The unfamiliar person tossed a treat to the puppy through the bars of the pen and recorded if they ate it or not.

Step 2: Open—The unfamiliar person, still turned to the side with averted gaze, opened the pen door and scored the puppy’s behavioral response. The unfamiliar person extended one hand and offered a treat to the puppy and recorded whether or not they ate it.

Step 3: Reach—With the same orientation, the unfamiliar person reached toward the puppy with one hand, recorded the puppy’s behavioral response, and if he/she could touch the puppy. Puppies were only touched if they were in the front half of the pen and oriented toward and/or interacting with the unfamiliar person. The unfamiliar person then retracted his/her hand, extended his/her other hand to offer the puppy a treat, and recorded if the puppy ate it or not.

The unfamiliar person produced auditory stimuli by tapping the pen door with his/her fingers or making soft “kissing” noises to elicit puppies’ attention at the beginning of each step. This was often necessary, as some puppies likely did not have full visual acuity when tested but had fully developed auditory startle responses [40]. 

Puppies’ behavioral responses to each step of the PuppyFIDO test were scored via video, using the ethogram outlined in Table 2.

#### 2.5.2. Isolation Test

Immediately following completion of the PuppyFIDO test, Researcher 2 picked up any uneaten treats from the pen, and the researcher walked out of the puppy’s sight, leaving them alone for the three-minute isolation test, which was timed by using a stopwatch. When three minutes had elapsed, Researcher 2 returned to the isolation pen and performed the postISO PuppyFIDO test (both the behavior and physical health portions). Upon conclusion, the puppy was returned to its littermates, and a new puppy was retrieved for testing.

Puppy behaviors analyzed during the isolation test are listed in Table 3. Behavioral video coding was conducted by Researcher 1, using the Behavioral Observation Research Interactive Software (BORIS) version 7.9.7 [43]. Six videos were dropped from analysis due to interruptions during the testing process, leaving 114 coded videos.

### 2.6. Data Analysis

An initial exploratory analysis was performed by using descriptive statistics and graphic visualizations, which were utilized to guide further statistical analysis. Means, standard errors of the means, percentages, chi-square tests, and basic graphs for visualization were created in Excel. Remaining analyses were performed in R version X (R Core Team, Vienna, Austria), utilizing the “nlme”, “lme4”, “car”, and “emmeans” packages with α ≤ 0.05 [49,50,51,52].

#### 2.6.1. Physical Health

Descriptive statistics were utilized to determine the percentage of puppies from each treatment group who showed signs of any physical health problems throughout the first eight weeks of life (i.e., at the breeder), at the breeder’s kennel on the day of behavioral testing, and at the distributor (i.e., after transport stressor).

Weight and weight gain were assessed for the first eight weeks, for the testing days at the breeder’s kennel, and at the distributor, using separate general linear mixed-effects models (GLMEs). The treatment group (ENS, held, and control), timepoint (week), sex, and breed were included in the model as fixed effects. All relevant interaction effects were also included. The cohort, litter, and puppy ID were included as nested random factors to account for non-independence of puppies across measurements, as well as litter and cohort effects. Model residuals were checked for normality and homoscedasticity, as per the assumptions of a GLME. A backward stepwise approach was used to remove interactions and factors and increase model fit, assessed via Akaike’s information criterion (AIC) values. Model fit was evaluated by using maximum likelihood (ML), and test statistics (χ^2^ and *p*) were extracted from the best-fitting model, using restricted maximum likelihood (REML) and a Wald’s test.

#### 2.6.2. Stranger Approach Tests

To evaluate whether the treatment group affected puppies’ responses to the PuppyFIDO test, scores were assigned for each step of the test as follows: Orientation (Yes = 1, No = 0), Approach (Approach = 2, Ambivalent Approach = 1, No Approach = 0), Behavior (Affiliative–Outgoing = 2, Affiliative = 1, Undisturbed = 0, Stationary = 0, Avoid = −1), Posture (Normal = 1, Low = 0), Treat (Yes = 1, No = 0), and Touch (Yes = 1, No= 0). These scores were summed to create an overall score (summed PuppyFIDO score, maximum points = 22). 

All videos were coded by one person who was blind to the experimental design (Researcher 2). The inter-rater reliability of this tool was calculated and reported in a separate study between the same coder (Researcher 2) and one other independent coder (SB). Agreement levels were moderate to perfect for all metrics (range = 0.60- 1) [2].

The summed PuppyFIDO scores were assessed via GLME. Each model contained treatment group, isolation (i.e., pre- or post-isolation), transport (i.e., pre- or post-transport), and ear-crop (1 = ear crop; 0 = no ear crop) as fixed effects, as well as the interactions between the three. Cohort, litter, and puppy ID were also included in these models as nested random effects. Normality and homoscedasticity of model residuals were evaluated, and a backward stepwise model selection approach using ML and AIC values were again used to indicate the best fitting model. The best fitting model was run with REML and the test statistics (χ^2^ and *p*) were extracted by using a Wald’s test. If fixed effects were statistically significant, a post hoc analysis was conducted.

#### 2.6.3. Isolation Test

All videos were scored by Researcher 1. The inter-rater reliability was calculated on 17 videos (15%) that were scored by a second independent observer who was blind to the experimental design. Intra-rater reliability was assessed via re-coding of 12 videos (9.5%) after four months by the same researcher. Agreement was calculated by using intraclass correlation coefficients (ICCs). ICC scores > 0.70 with a *p*-value < 0.01 were considered acceptable for reliability purposes. 

Means, standard deviations (SDs), and standard errors of the means (SEMs) were calculated for the behaviors scored during the 3-minute isolation tests, and cross-tabulations were used to identify the most common behaviors (longest duration) for each treatment group. Self-grooming, repetitive behaviors, shaking off, and elimination were dropped due to low occurrence. Each behavior was compared by using separate GLMEs, which included the treatment group and transport (i.e., pre- or post-transport), along with their interaction as fixed effects, and the nested random effects of cohort, litter, and puppy ID. Model residuals were checked for normality and homoscedasticity. A backward stepwise model-selection method, using AIC values, was again used to increase model fit. Models were fitted by using ML, and the test statistics (χ^2^ and *p*) were extracted from the best-fitting models, using a Wald’s test and REML. 

## 3. Results

### 3.1. Physical Health

**Figure 3:**
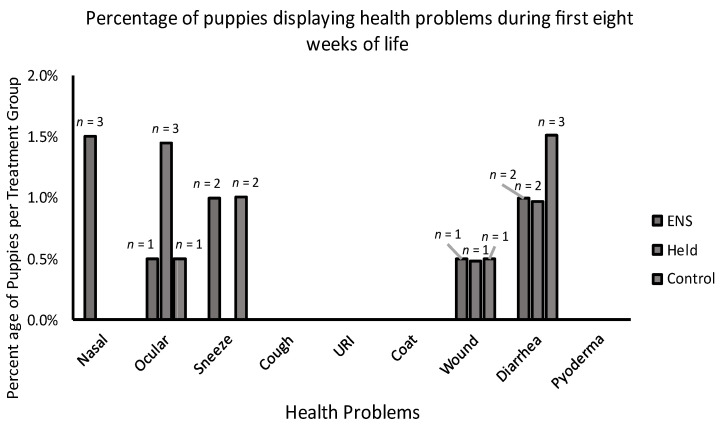
Percentage of puppies in each treatment group (ENS, held, and control) showing each physical health problem measured across the first eight weeks of life prior to the application of stressors.

Over the first eight weeks of life (i.e., at the breeder’s kennel) and prior to the application of stressors (i.e., isolation testing and transport to the distributor), only 3.3% of all puppies showed evidence of any physical health problems (*n* = 20). Percentages of puppies showing each physical health problem by treatment group are shown in Figure 3.

Over the first eight weeks of life and prior to the application of stressors, 92.4% of puppies were scored as “clean”, with less than 25% of their bodies covered in debris. A small number of puppies (*n* = 19) was scored as 26–50% “debris-covered” for at least one time point; however, 11 of these puppies were scored 26–50% at just a single time point during this period. Only eight puppies were scored 26–50% on more than one assessment over this time. No puppies were scored higher than 50%. During the transport week, most puppies (93.4%) were scored as “clean”. Eight puppies were scored as 26–50% “debris-covered” at the breeder’s kennel pre-transport; however, these puppies were all scored as clean or <25% at the distributor post-transport.

Using our novel three-point body-condition score, all puppies were scored as having a “normal” body condition.

A small wound was noted for one puppy from the ENS group and one from the control group at the breeder’s kennel, two to three days pre-transport. At the distributor 48 h post-transport, a different puppy from the control group was observed to have a cough, and another was found to have a minor wound.

### 3.2. Weight and Weight Gain

**Figure 4:**
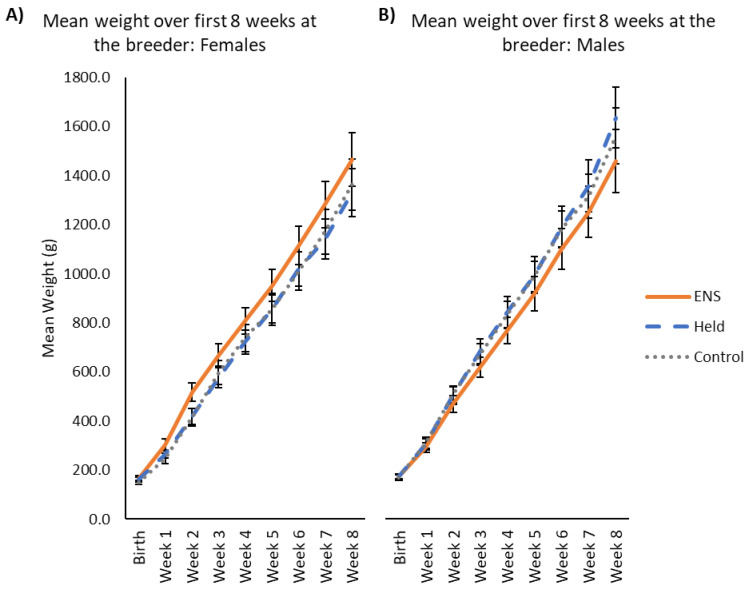
Mean weight for (**A**) female and (**B**) male puppies in each treatment group over the first eight weeks at the breeder’s kennel, prior to application of stressors. Error bars represent the standard errors of the means.

Puppy weight gain was significantly affected by week (χ^2^ = 6560.73; *p* < 0.001) and breed (χ^2^ = 20.15; *p* = 0.001) over the first eight weeks. Weight also varied by a three-way interaction between treatment group, week, and sex (χ^2^ = 10.21; *p* = 0.006). Descriptively, female puppies in the ENS group consistently weighed more than their held and control-group counterparts, while held and control-group male puppies were observed to be heavier than males in the ENS group, starting around two weeks of age. At around seven weeks of age, male puppies in the held group began to weigh more than control-group males (Figure 4).

Neither the treatment group nor any of its interactions had a significant effect on weight *gain* over the first eight weeks of life. Puppy weight gain was, however, significantly affected by week (χ^2^ = 26.71; *p* < 0.001), sex (χ^2^ = 5.62; *p* = 0.02), and breed (χ^2^ = 23.74; *p* < 0.001).

### 3.3. PuppyFIDO

**Figure 5:**
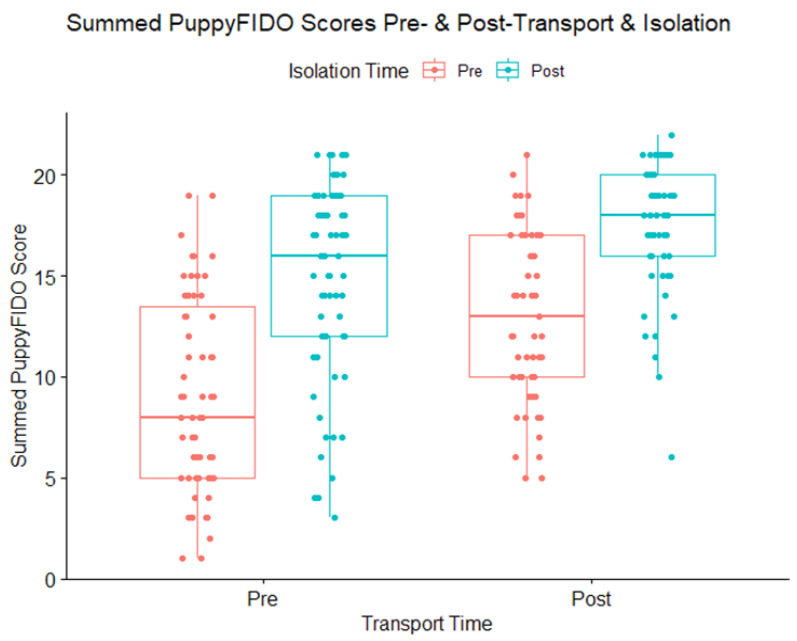
Summed scores for puppies tested with the PuppyFIDO tests pre- and post-isolation (isolation time) and transport to the distributor (transport time). Higher scores represent more affiliative behaviors in response to a stranger approaching.

Isolation and transport both had significant effects on puppies’ behavioral responses to a stranger’s approach (Figure 5). Puppies displayed more affiliative behaviors post-isolation than pre-isolation, regardless of whether the test occurred before or after transportation (χ^2^ = 145.31; *p* < 0.0001). Puppies also displayed more affiliative behaviors at the distributor post-transport than at the breeder’s kennel pre-transport (χ^2^ = 64.09; *p* < 0.0001). The treatment group did not have a significant effect on puppies’ behavioral responses to a stranger’s approach (χ^2^ = 0.27; *p* = 0.87), and there were no significant interactions between treatment, isolation, and transport. 

**Figure 6:**
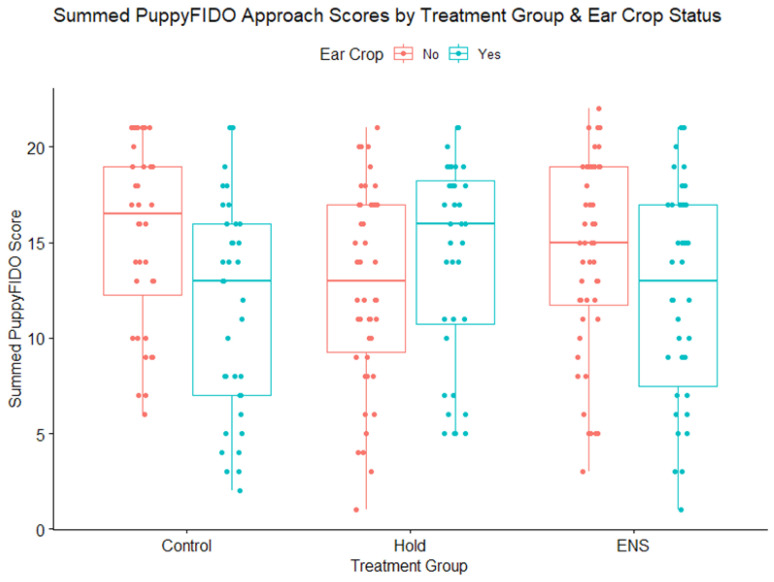
Summed scores for puppies from the PuppyFIDO test showing differences between the three treatment groups (control, held, and ENS) for puppies with cropped ears (Yes) and uncropped puppies (No). Higher scores represent more affiliative behaviors in response to stranger’s approach.

The ear croppedand treatment group showed a significant interaction effect (χ^2^ = 7.00; *p* = 0.03). The post hoc analysis, however, returned no significant value for pair comparisons. Descriptively, summed PuppyFIDO scores for puppies whose ears were cropped were lower (i.e., less affiliative) in both the control (mean = 11.67; SE= 0.93) and ENS (mean = 12.29; SE = 0.90) groups than in those whose ears were uncropped (control: mean = 15.39, and SE = 0.79; ENS: mean = 14.27, and SE = 0.74). In the held group, however, PuppyFIDO scores were higher (i.e., more affiliative) for puppies with cropped ears (mean = 14.14; SE = 0.89) than those with unaltered ears (mean = 12.89; SE= 0.78) (Figure 6).

### 3.4. Isolation

Inter-rater reliability was moderate to excellent (ICC range 0.74–0.96; *p* < 0.01) for all behaviors assessed. Intra-rater reliability was also moderate to excellent (ICC range 0.87–0.96; *p* < 0.01) for all comparisons.

The treatment group did not significantly affect the amount of time puppies spent performing any of the behaviors measured during the isolation test. Transportation had a significant effect on three behaviors: exploratory locomotion, standing, and vocalization. Puppies displayed shorter durations of exploratory locomotion (χ^2^ = 26.06; *p* < 0.0001), longer durations of standing behavior (χ^2^ = 38.62; *p* < 0.0001), and greater frequencies of vocalizations (χ^2^ = 6.40; *p* = 0.011) at the distributor post-transport than at the breeder pre-transport. 

## 4. Discussion

Based on previous studies of ENS in dogs and other animals [12,13,17,19,33,36,53], we hypothesized that puppies who received ENS would exhibit indications of improved physical health, such as less illness and increased growth, as compared to puppies who were simply held, or who received no early handling treatments. Puppies in the ENS treatment group were also expected to demonstrate increased affiliative behavior toward strangers, as well as less fear and more exploration of a novel environment when socially isolated, when compared to held and control-group puppies.

### 4.1. Physical Health

The first hypothesis, that puppies who received ENS would be physically healthier than those in the held or control groups, was not supported. Each treatment group showed very few health problems over the first eight weeks of life (control = 3.51%, held = 2.89%, and ENS = 4.5%), so it was not possible to statistically evaluate the effects of treatment on puppies’ physical health. Furthermore, most puppies were found to be clean (i.e., absent of debris) throughout the entire study. Puppies’ body cleanliness was evaluated as a proxy measure for management factors, which can affect dog welfare, such as kennel-cleaning protocols or pen sizes [54]. However, our data showed that, among the puppies who were scored to have 26–50% of the body with signs of debris across multiple time points, all but one came from the same litter. This suggests differences in maternal-care quality rather than treatment or kennel management. These findings indicate that most puppies had good physical health, which is consistent with previous investigations in puppies and adult dogs in USA CB kennels [2,55].

### 4.2. Weight and Weight Gain

The only area where some puppies in the ENS group showed changes in physical health metrics was weight. While previous studies of ENS in dogs have not demonstrated effects on weight or weight gain e.g., [33] a three-way interaction between treatment, sex, and week was observed in the current study, with female puppies in the ENS treatment group consistently weighing more than their held and control counterparts. In contrast, male puppies in the held and control groups weighed more than those in the ENS group. It is unclear why treatment had opposite effects on weight in male and female puppies, and it is possible the finding was unique to this sample. Despite differences in weight between treatment groups, all puppies had a normal BCS, providing additional evidence of good physical health.

### 4.3. PuppyFIDO

The hypothesis that puppies who received ENS would be more affiliative toward strangers was not met, as there were no significant differences between treatment groups in response to stranger approach. One possible explanation is that all puppies in the study received enough positive interactions with people during their first eight weeks of life to mask the effects of additional handling from ENS or held treatments, similar to Schoon and Berntsen’s observations in their study of the Bio Sensor program [35]. This could be a result of standard interactions with caretakers since normal kennel procedures were continued for all puppies in this study. It could also be an artifact of the study design because all puppies were marked daily for identification purposes and subjected to a hands-on physical health assessment weekly. Furthermore, as we did not see an interaction effect between treatment and transport, it may be that the cumulative effect of stressors involved with transport (i.e., bathing, grooming, handling by multiple unfamiliar people, and moving to a novel environment) was so profound that a ceiling effect occurred, and testing was unable to elucidate differences between treatment groups. However, this is unlikely, as no differences between treatment groups were observed pre-transport. It is important to note that there was a significant interactive effect between ear cropping and treatment group on puppies’ overall PuppyFIDO test scores, but the post hoc pair comparisons were not statistically significant. Nevertheless, mean values showed that puppies in the control and ENS groups who had their ears cropped displayed fewer affiliative behaviors than those who did not, but for the held group, puppies who had been cropped showed more affiliative behaviors. It can be hypothesized that less intense types of neonatal handling (i.e., more positive interactions experienced with people) may be more effective for those puppies who have been subjected to aversive early experiences. The impact of these invasive procedures on puppies’ stress susceptibilities warrants further investigation, as associations between invasive procedures such as tail docking and response to an unfamiliar person later in life have also been observed in piglets [56].

Puppies’ interactions with the researcher during handling treatments or weekly physicals, or with caretakers during normal kennel interactions or daily marking, may have primed them to view people as social buffers during stressful experiences. If they perceived the stressor of isolation as more aversive than being approached by a stranger, this could explain why puppies displayed more affiliative behaviors toward the unfamiliar person post-isolation testing, which was also observed in a similar study by Romaniuk et al. [2]. This explanation would be consistent with existing knowledge that early experiences shape development, which is one of the underlying principles used to support theories of ENS, as discussed previously, e.g., in [10,22]. Furthermore, it is possible that the change in responses from pre- to post-transport reflected that the puppies experienced more distress after transportation than before and therefore sought comfort from the unfamiliar person, as outlined by Romaniuk et al. [2]. The changes in behaviors exhibited during the isolation test at the distributor support this explanation and are discussed below.

### 4.4. Isolation

Our next hypothesis, that puppies who received ENS would display less fear-related behaviors and more exploratory behaviors when isolated, was also not met. However, transport did have a significant effect on the behaviors measured during isolation. Regardless of treatment, puppies spent significantly less time performing exploratory locomotion and more time standing at the distributor than at the breeder. They also vocalized more at the distributor post-transport than at the breeder pre-transport. These behaviors are likely indicative of distress [57,58] associated with arrival at a novel environment (i.e., the distributor) and/or residual effects of transport stress. These findings are consistent with Romaniuk et al. [2], as they also found that puppies exhibited increased durations of behaviors likely indicative of distress following transportation to a distributor, along with increases in cortisol concentrations and measures of immune function. 

### 4.5. Limitations

Some limitations of the current study exist. A more comprehensive welfare assessment including physiological metrics, such as cortisol concentrations, heart rate, or heart rate variability, in addition to the health and behavioral metrics collected, would have allowed for a better understanding of stress levels puppies may have experienced throughout the study. In addition, marking individual puppies for identification proved quite challenging, due to dams removing the marks when cleaning their puppies. Long-term colorings such as livestock paint were not used because they could not be removed without shaving the puppies’ hair, which would have negatively impacted their appearance and ability to be sold to the public. The short-term durability of marking crayons and pens used therefore required all puppies to be handled briefly each day to be re-marked. This introduced slightly more handling for each puppy than originally intended, but since the process was the same for all, differences between treatment groups regarding total amounts of handling were maintained. However, the overall increase in handling may have masked some effects of treatment, as previously noted. Furthermore, data in the current study were collected on a limited number of breeds, most of which had small litter sizes (<5), and all of them were all raised in a single kennel. Therefore, the results of this study cannot be generalized to the entire CB kennel population, as there may have been effects of different breeds or routine handling procedures in the test kennel that could have been sufficient to produce positive effects for all puppies.

### 4.6. Future Directions

On the basis of our results, future studies should explore the impacts of invasive procedures experienced early in life, such as ear cropping, on puppies’ stress susceptibilities later in life and how ENS may modulate these effects. Furthermore, future studies should explore how various management practices may influence the effects of ENS on puppy welfare in CB kennels. Since the most recent study examining Bio Sensor ENS did not find differences between ENS and control-group puppies until 10 to 12 months of age [37], future studies of ENS should attempt to follow dogs for similar time periods to identify if differences between treatment groups emerge later in life. Finally, there may have been a ceiling effect of transportation stress masking the effects of ENS. Measuring stress levels and giving the puppies more time to adapt to the new environment might have revealed group differences in recovery time and coping strategies.

## 5. Conclusions

This study aimed to determine the effects of the Bio Sensor ENS on the welfare of puppies from CB kennels. The findings indicated that ENS neither improved puppies’ physical health (due to very good overall health from the outset) nor decreased their negative responses to stress as purported. These results are supported by the other two published studies which have evaluated the Bio Sensor program [35,37], but they differ from Battaglia’s original report [34]. Independent of treatment, puppies may have perceived an unfamiliar person as a potential social buffer during stressors. Collectively, from these results, the welfare benefits of applying Bio Sensor ENS exercises to puppies raised in CB kennels were not confirmed. Future studies with larger sample sizes that follow dogs further into their lives and consider their early life experiences are needed.

## Figures and Tables

**Table 1:** Breeds represented in the sample, number of puppies per breed, and number of puppies per treatment group within each breed.

Breed	Number of Puppies	Control	ENS	Held
Bichon/Toy Poodle cross	8	3	2	3
Miniature Pinscher *	13	4	5	4
Miniature Schnauzer *	16	6	5	5
Pomeranian	15	6	4	5
Bichon/Shih Tzu Cross	11	3	4	4
Toy Poodle	13	3	5	5
Total	76	25	25	26

* Received ear crops between two and six weeks of age.

**Table 2:** Ethogram of puppy behaviors used to score their behavioral response to each step of the stranger-approach test [2].

Stage	Response	Definition
Orientation	Orientation	The puppy acknowledges the experimenter (i.e., makes eye contact/ is oriented toward) within 7 s.
	No Orientation	The puppy does not acknowledge the experimenter’s presence within 7 s (or the duration of the step).
Approach	Approach	The puppy moves toward the experimenter (i.e., takes steps toward him/her or leans toward him/her if he/she cannot step any closer).
	Ambivalent Approach	The puppy approaches and retreats or approaches but then stops before reaching the experimenter [38].
	No Approach	The puppy does not approach (i.e., does not move toward the experimenter) [38].
Behavior	Affiliative Behavior	Any behaviors exhibited by the puppy that are intended to facilitate the establishment or reinforcement of a social bond. Examples include approaching the experimenter while maintaining eye contact and/or making physical contact (e.g., licking and touching) with the experimenter. Outgoing The puppy jumps up or “scrambles” at the front of cage and/or attempts to cross/crosses the front barrier of cage and/or exhibits repeated physical contact with the experimenter (e.g., repeatedly licking, jumping on hands, etc.) and/or approaches the experimenter while exhibiting intense tail wagging.
	Undisturbed	The puppy is engaging in an active behavior (e.g., sniffing, eating, etc.) when the step begins, then acknowledges the experimenter’s presence and returns to the same behavior [38].
	Avoid	The puppy avoids the experimenter (i.e., moves away from them and turns its head in the opposite direction) [38].
	Stationary	The dog is in a static posture (i.e., sitting and lying). There may or may not be visual orientation toward the environment. The dog may change posture in place but does not show any displacement [41]
Posture	Normal	“Normal posture under neutral conditions” for specific breed and age [42]
	Low	“Back rounded and/or legs bent…, head lowered” [42].
Additional	Fight/ Aggression	The puppy exhibits aggression (e.g., lunging, growling, teeth baring, etc.) [38].
	Bark	Negative affect Barking associated with avoidance, aggression, frustration, etc. Positive affect Barking associated with play, greeting, excitement, etc.
	Stereotypic Behavior	The puppy performs a pattern of behavior repeatedly (e.g., pacing, circling, etc.) [38].

**Table 3:** Ethogram used for isolation video scoring.

Category	Behavior	Description
Locomotory behaviors	Exploratory locomotion (duration)	Puppy engaged in motor activity (e.g., walking, trotting, and running) involved in targeted investigation of the environment (sniffing, pawing, licking, etc.) (adapted from [36,44]).
	Non-exploratory locomotion (duration)	Puppy engaged in motor activity (e.g., walking, trotting, and running) without exploration of the environment (adapted from [44]).
	Repetitive behaviors * (duration)	Movement repeated along the same path (imaginary line, along the fence, or in a circle) or any stereotypic behaviors (repeated bouncing off pen walls, jumping up and down on the same spot with 2 or 4 legs in the air, pivoting on hind legs) (adapted from [45,46]).
Stationary behaviors	Sit (duration)	Front legs straight, rear end lowered, and resting on “hocks” and “perineum” or floor (adapted from [42,47]).
	Stand (duration)	Puppy is in an upright position supported by 3 or 4 legs (adapted from [46,47]).
	Lie down * (duration)	Puppy’s body is in contact with the ground, not supported by its legs. Head may be lifted or resting on the ground, and the eyes may be open or closed (adapted from [42,46]).
	Stationary exploration (duration)	Targeted investigation of the environment (sniffing, pawing, licking, etc.) while in a stationary position (sit, stand, or lie).
	Self-grooming * (duration)	“Action of cleaning of the body surface by licking, nibbling, picking, rubbing, scratching, and so on directed toward the animal’s body” [44].
	Shaking head/body * (frequency)	Rotation of the head or the entire body, beginning with the head and moving toward the tail [47].
	Escape attempt (duration)	Puppy attempts to leave test pen by pawing at pen walls, sticking nose through pen bars, or biting pen bars (adapted from [48]).
Vocalizations	Any vocalization, e.g., bark, growl, howl, or whine (frequency)	Bark: “Sharp vocalization, often loud and repetitive” Growl: “Low-pitched grumble, with or without exposed teeth” Howl: “Low pitched, long duration vocalization” Whine: “High-pitched vocalization” [42].
Elimination *	Urination (frequency)	Puppy expels urine from the body.
	Defecation (frequency)	Puppy expels feces from the body.

* Dropped from analysis due to low occurrence of behavior.

## Data Availability

Data are available upon request to the corresponding author.
